# Multi-omics profiling reveals the role of 4-ethylbenzoic acid in promoting proliferation and invasion of cervical cancer

**DOI:** 10.3389/fmed.2025.1591531

**Published:** 2025-10-13

**Authors:** Xiaoling Huang, Shan Lu, Xiaoge Li, Jin Wu, Qiao Zu, Zhaoning Duan, Ming Luo, Ying Jia

**Affiliations:** ^1^Chongqing Key Laboratory of Molecular Oncology and Epigenetics, Department of Obstetrics and Gynecology, The First Affiliated Hospital of Chongqing Medical University, Chongqing, China; ^2^Department of Obstetrics and Gynecology, Zhengzhou Central Hospital, Zhengzhou, China

**Keywords:** cervical cancer, 4-ethylbenzoic acid, metabolomics, proteomics, biomarker

## Abstract

**Background:**

Cervical cancer (CC) is a global health challenge, ranking fourth among cancers in women. Microbiome–metabolome interactions influence human papillomavirus (HPV) associated carcinogenesis, but specific microbial metabolites driving malignant progression remain undefined. This study aimed to identify potential biomarkers for distinguishing CC, and further explore their role in the progression of CC.

**Methods:**

Non-targeted metabolomics was employed to profile alterations in the vaginal microenvironment across clinical cohorts, including individuals with CC, individuals with cervical intraepithelial neoplasia (CIN), HPV-positive individuals, and HPV-negative individuals. Targeted metabolomics was then used to confirm the expression of 4-ethylbenzoic acid (4-EA) levels and its role in CC was explored using cell counting kit-8, 5-ethynyl-2′-deoxyuridine, colony formation, transwell, and wound healing assays. Proteomics was used to investigate the effects of 4-EA on CC cells.

**Results:**

The metabolic profiles of vaginal secretions in the CC group differed significantly from those in the other three groups. Untargeted metabolomics identified 27 CC-specific metabolites (VIP > 2, *p* < 0.05), revealing a marked elevation of 4-EA and its close relationship with vaginal microorganisms. Clinico-pathological correlations revealed progressive 4-EA accumulation across the cervical carcinogenesis stages. Additionally, 4-EA promoted the proliferation, migration, and invasion of CC cells *in vitro*. Proteomic reprogramming of CC cells following 4-EA treatment identified 14 highly expressed proteins associated with poor prognosis.

**Conclusion:**

This multi-omics investigation identified 4-EA as a novel candidate metabolite and a potential biomarker of CC. Identification of key proteins may provide new insights for interventions targeting the development of CC.

## 1 Introduction

Cervical cancer (CC) is the fourth most prevalent malignancy among women worldwide, with 661,021 new cases and 348,189 deaths in 2022 (1). Notably, China accounts for 22.8% of this disease burden, reflecting both rising incidence rates and a concerning demographic shift toward younger populations (2, 3). The occurrence of CC is a multifactorial process involving dynamic interactions within the cervicovaginal microenvironment—a complex ecosystem comprising host epithelial cells, immune mediators, microbiota, and metabolites. This microenvironment is dominated by *Lactobacillus* species under physiological conditions, which maintain vaginal health through lactic acid production (pH ≤ 4.5), bacteriocin secretion, and competitive exclusion of pathogens (4). A persistent high-risk human papillomavirus (HR-HPV) infection is a key factor in the development of CC (5). Additionally, metabolites derived from the vaginal microbiota are closely linked to HPV infection and cervical lesion progression (6, 7). Microbiome perturbations (*Lactobacillus* depletion and enrichment of *Prevotella, Gardnerella, Sneathia*) alter metabolic outputs—including biogenic amines, short-chain fatty acids, and microbial derivatives—which modulate epithelial barrier integrity, local inflammation, and HPV persistence (7, 8).

Metabolic reprogramming, a hallmark of malignancy (9), recognizes metabolites as functional mediators of carcinogenesis and potential diagnostic targets. Metabolomics has emerged as a powerful tool for identifying cancer biomarkers owing to its capacity to capture real-time biochemical activity (10, 11). Emerging research emphasizes the functional roles of metabolites in physiology and diseases. For example, *α*-ketoglutarate regulates macrophage immune responses (12), and metabolites such as phospholipids and amino acids regulate insulin sensitivity (13). Furthermore, lysophosphatidylcholine inhibits lung cancer proliferation by inducing mitochondrial dysfunction and altering lipid metabolism (14). Specifically, in CC, C8 ceramide-1-phosphate exerts tumor-suppressive effects through the MAPK/JNK1 pathway (15).

HR-HPV infection remodeling of the vaginal microenvironment, results in a self-perpetuating cycle of dysbiosis and metabolic dysregulation (7, 16). CC-associated metabolomic signatures exhibit profound alterations in amino acid, lipid, and carbohydrate pathways (17, 18); however, the mechanistic contributions of individual metabolites remain unclear. Current studies predominantly catalog metabolic shifts without bridging correlative observations to functional validation, which is a critical gap hindering clinical translation.

In this study, we mapped the cervicovaginal metabolic profile throughout cervical carcinogenesis using liquid chromatography-mass spectrometry (LC–MS). 320 longitudinally vaginal lavage samples were collected, and stratified into cervical cancer (CC), cervical intraepithelial neoplasia (CIN), HPV-positive, and HPV-negative cohorts. We then validated the role and potential mechanisms of these identified metabolites in CC development through *in vitro* experiments and proteomics. By establishing a vaginal microbiome-metabolite-key protein network, this study provides a novel theoretical framework for early CC detection and lays the foundation for subsequent mechanistic exploration of oncogenic pathways.

## 2 Materials and methods

### 2.1 Participants

A total of 320 vaginal lavage samples were collected from female patients undergoing gynecological examinations for non-targeted metabolomics analysis at the First Affiliated Hospital of Chongqing Medical University in China from January 2021 to May 2021. The characteristics of the patients and their demographic information have been previously detailed (19). This study was approved by the Ethics Committee of the First Affiliated Hospital of Chongqing Medical University (Ethics NO. 2023-24). Based on the HPV test, ThinPrep liquid-based cytology test (TCT), and biopsy pathology, the participants were classified into four groups: cervical cancer group (CC) group, HPV-positive with cervical intraepithelial neoplasia group (CIN), HPV-positive without cervical lesions [HPV (+)] and HPV-negative healthy control group [HPV (−)], with 80 cases in each group. The TellgenplexTM HPV DNA (real-time PCR) Test (Tiansheng Biotech Co., Ltd., Shanghai, China), targeting the L1 gene, was utilized for 14 HR-HPV subtypes (16, 18, 31, 33, 35, 39, 45, 51, 52, 56, 58, 59, 66, and 68) assay. For histopathological diagnosis, two gynecologic pathologists independently reviewed 4 μm formalin-fixed paraffin sections stained with hematoxylin–eosin (H&E), in accordance with the WHO 2020 classification guidelines. The inclusion and exclusion criteria are previously described (19). In short, participants in this study included individuals aged 20 years and older who were sexually active, and provided written informed consent. Specifically, pregnant, breastfeeding, or menstruating females; those who had sexual intercourse or vaginal lavage within the past week; those who have used antibiotics within the past month; those receiving long-term hormones or immunosuppressants therapy; or those who have undergone cervical surgery were excluded. Additionally, 32 vaginal lavage samples were collected for targeted metabolomic detection following the same inclusion and exclusion criteria and classification into four groups. Patient characteristics and demographic information are provided in Supplementary Table 1.

### 2.2 Vaginal lavage sample collection for metabolomics analysis

A sterile speculum was used to expose the vagina, and a suitable amount of sterile physiological saline was used to clean the cervix and upper part of the vagina. Approximately 5 mL of the lavage fluid was collected and stored at ˗80 °C for subsequent metabolomics analysis.

### 2.3 Vaginal microbiome analysis

Vaginal microbiome analysis was performed as previously described (17). Briefly, vaginal secretion samples were collected using sterile swabs. After DNA extraction, PCR amplification, and library construction, the PCR products were sequenced on the Illumina® MiSeq platform (San Diego, California, USA). Bioinformatics analysis of the 16S rDNA sequencing data was conducted using a custom QIIME2 software pipeline (University of Colorado Boulder, Boulder, CO, USA).

### 2.4 Non-targeted metabolomics analysis

#### 2.4.1 Metabolite extraction

Vaginal lavage samples from 320 patients were stratified into four cohorts (*n* = 80 per group). Within each group, samples were pooled into composite sets (*n* = 20 samples/set) for aggregate metabolomic profiling. Pooled samples were vortex-mixed for 10 min, followed by metabolite extraction where 300 μL aliquots were combined with 900 μL of methanol and acetonitrile mixture (v/v, 1:1). After vortexing (1 min) and centrifugation (17,000 g, 15 min, 37 °C), the supernatant was diluted with 80 μL of 50% (v/v) acetonitrile and recentrifuged under identical conditions. The resulting supernatant was stored at −80 °C for UPLC–MS/MS analysis, with quality control (QC) samples generated by pooling 10 μL aliquots from each sample.

#### 2.4.2 Ultra-performance liquid chromatography–tandem mass spectrometry (UPLC–MS/MS) analysis

Chromatographic separation was achieved using an UltiMate 3,000 UPLC–MS/MS system (Thermo Fisher, Waltham, MA, USA) coupled to an AB SCIEX 5600 Triple TOF mass spectrometer (AB SCIEX, Framingham, MA, USA). Separations utilized an ACQUITY UPLC HSS T3 column (2.1 × 100 mm, 1.8 μm, Waters, Milford, MA, USA) with a 400 μL/min flow rate. Mobile phases consisting of solvent A (0.1% aqueous formic acid and 0.1% formic acid in acetonitrile), and solvent B (2 mM ammonium acetate and pure acetonitrile). The gradient elution program initiated with 5% B (0–1.5 min); 5–10% B for 1.5–2.5 min; 10–40% B for 2.5–14.0 min; 40–95% B for 14.0–22.0 min; 95% B for 22.0–25.0 min. The mobile phase was then adjusted to initial conditions (5% B) within 1 min and equilibrated for 4 min. Mass spectrometric parameters included dual electrospray ionization (ESI±) with voltages set to 5.0 kV (positive) and 4.0 kV (negative). The ion source operated at 650 °C, supported by dual gas pressure (GS1/GS2: 60 psi each) and a 35 psi curtain gas pressure.

#### 2.4.3 Data analysis

The converted ABF file was processed using MDIAL 4.24 software (RIKEN Center for Sustainable Resource Science, Wako, Japan), including peak searching, peak alignment, removal of blank values, and identification of results obtained. The parameters for the MSDIAL software were established as follows: MS1 tolerance: 0.01 Da; Retention time tolerance: 0.2 min; Accurate mass tolerance (MS1): 0.01 Da; (MS2): 0.05 Da. Identification score cut off: 60%. All features detected in QC samples (*n* = 6 injections) required CV < 30% across replicates, and identified metabolites were further verified for CV < 30%. Peaks intergroup missing values >50% were excluded. Normalization was performed using the total ion current and differential and clustering analyses were conducted using MetaboAnalyst 6.0 software (McGill University, Montreal, QC, Canada). Differential metabolites were identified through a two-tiered approach: variables with VIP > 1.0 (*p* < 0.05) from orthogonal partial least squares-discriminant analysis (OPLS-DA) models were retained for initial screening. And a stricter threshold of VIP > 2.0 (*p* < 0.05) was applied to prioritize metabolites with the highest discriminatory power for downstream validation.

### 2.5 Targeted metabolomics

#### 2.5.1 Sample and standard curve construction

A standard 4-ethylbenzoic acid (4-EA) stock solution (1 mg/mL) was diluted with a 75% methanol solution to a specific concentration to prepare the standard working solution. A standard working curve was constructed using a standard solution.

#### 2.5.2 Metabolite extraction

Following thawing at 4 °C, aliquots (100 μL) were mixed with methanol in a 1:3 ratio (v/v), homogenized by vortexing for 60 s, and centrifuged at 17000 *g* for 15 min to collect clarified extracts for subsequent analysis.

#### 2.5.3 UPLC–MS/MS analysis

Chromatographic separation was performed on an ACQUITY UPLC BEH Amide column (3.0 × 100 mm, 1.7 μm; Waters, Milford, MA, USA) using an ACQUITY UPLC system coupled with an AB 4500 triple quadrupole mass spectrometer(AB SCIEX, Framingham, MA, USA). Detection was conducted in positive ionization mode using a mobile phase consisting of solvent A (10 mM ammonium acetate with 0.1% formic acid in water) and solvent B (90% acetonitrile with 10 mM ammonium acetate and 0.1% formic acid). Solvent A multistep gradient protocol was implemented: 5% A (0–5 min), 5–30% A (5–7 min), 30–80% A (7–10 min), 80–95% A (10–12 min), with rapid re-equilibration to 5% A within 0.1 min and stabilization for 3 min. Mass spectrometric detection in multiple reaction monitoring(MRM) mode utilized optimized parameters: ion source and nebulizer temperatures at 500 °C, curtain gas 25 psi, collision gas 10 psi, and ion spray voltage 4,500 V.

#### 2.5.4 Data analysis

Using MultiQuant3.0.3 analysis software (AB SCIEX Pte. Ltd., Singapore), the response of the standard solution at known concentrations was used to construct a standard curve to calculate the sample concentration.

### 2.6 *In vitro* experiments

#### 2.6.1 Cell culture

CC cell lines HeLa and SiHa (Procell Life Science & Technology Co., Ltd., Wuhan, China) were purchased and maintained in DMEM (Gibco; Thermo Fisher Scientific, New York, USA) supplemented with 10% fetal bovine serum (FBS) (Gibco) and 1% penicillin–streptomycin (Gibco), and incubated at 37 °C with 5% CO2. The cells were passaged or seeded using 0.05% trypsin (GenView Co., Shanghai, China) upon reaching 80–90% confluence. All cell lines were maintained within 10 passages and underwent short tandem repeat (STR) profiling (17 loci) with quarterly mycoplasma testing via PCR amplification.

#### 2.6.2 Cell treatment

4-EA (Sigma-Aldrich, St. Louis, MO, USA) was dissolved in DMSO as a stock solution of 100 μM, aliquoted, and stored at −20 °C. Working concentrations were freshly diluted in antibiotic-free medium, with controls matching DMSO concentrations (≤0.1% v/v) to account for solvent effects.

#### 2.6.3 Cell counting kit-8 (CCK-8) cell proliferation assay

HeLa and SiHa cells in the logarithmic growth phase were seeded in 96-well plates (3,000 cells/well). After 24 h incubation at 37 °C in a 5% CO₂, cells were treated with varying concentrations (0 nM, 5 nM, 50 nM, 100 nM) of 4-EA. Then incubated with CCK-8 reagent (APExBIO, Shanghai, China) for 1.5 h, and absorbance at 450 nm was quantified using a microplate reader.

#### 2.6.4 Colony formation assay

Single-cell suspensions (HeLa or SiHa cells, 500 cells/well) were seeded into 6-well plates and treated with 50 nM 4-EA for 24 h attachment. Colonies were allowed to develop for 10–14 days, and the medium was renewed after every 3–5 days. Visible colonies (>50 cells) were fixed with 4% paraformaldehyde (PFA), stained with 0.1% crystal violet, and quantified using ImageJ software.

#### 2.6.5 5-ethynyl-2′-deoxyuridine (EdU) proliferation assay

Cell proliferation was assessed using the BeyoClick™ EdU-555 Kit (Beyotime Co., Shanghai, China). Briefly, HeLa (1 × 10^5^) or SiHa (3 × 10^5^) cells were treated with 50 nM 4-EA for 48 h, pulsed with 10 μM EdU (37 °C, dark), fixed with 4% PFA, and permeabilized with 0.3% Triton X-100. The cells were incubated with the reaction system, and the nuclei were counterstained with Hoechst 33342 (1:1000). Images were acquired using fluorescence microscopy and analyzed using the ImageJ software.

#### 2.6.6 Wound healing assay

HeLa and SiHa cells were seeded into 6-well plates and cultured overnight. Confluent monolayers were scratched using sterile 200 μL pipette tips, washed with phosphate-buffered saline, and cultured in serum-free DMEM containing 0 or 50 nM 4-EA. Migration progression was documented through time-lapse imaging (at 0, 24, 48 h). The scratch area was calculated by ImageJ software. Migration rates were calculated as follows: healing rate (%) = (initial area − residual area)/initial area × 100%.

#### 2.6.7 Transwell migration and invasion assay

Cells were pretreated with 0 or 50 nM 4-EA for 48 h before seeding into the chamber. For the migration assay, 200 μL of cells (1 × 10^5^ cells/mL) in FBS-free medium were loaded into upper chambers (Labselect Co., Beijing, China), with 600 μL 20% FBS as chemoattractant. After 24 h (HeLa cells) or 36 h (SiHa cells) of incubation, the migrated cells were fixed with 4% PFA, stained with 0.1% crystal violet, and counted using ImageJ software. For the invasion assay: Matrigel-coated chambers (Labselect Co.) were hydrated with prewarmed medium (500 μL, 2 h, 37 °C) before cell seeding. Following 24–36 h of incubation, the transmigrated cells were fixed, stained and counted as described above.

#### 2.6.8 Statistical analysis

Graphs were generated using GraphPad Prism 8.0 (GraphPad Software, LLC, San Diego, CA, USA), for two-group comparisons, the Student’s t-test was utilized, and for multiple groups, *p*-values were determined by one-way analysis of variance (ANOVA) with Tukey’s *post hoc* correction. The final figure assembly in Adobe Illustrator 2021. Significance thresholds set at *p < 0.05, **p < 0.01, and ***p < 0.001.

### 2.7 Proteomic analysis

#### 2.7.1 Protein sample preparation

HeLa cells were treated with 50 nM 4-EA for 48 h following overnight seeding. Total cellular proteins were extracted using RIPA lysis buffer (Beyotime Co., Shanghai, China) and quantified using a BCA protein assay kit (Beyotime Co.). Aliquots containing 20 μg protein were reduced with 100 mM tris(2-carboxyethyl) phosphine (Sigma-Aldrich) in 50 mM ammonium bicarbonate (56 °C, 60 min), then alkylated using 200 mM chloroacetamide (freshly prepared) in the dark (25 °C, 30 min). Trypsin digestion (Sigma-Aldrich) was performed overnight at 37 °C using 50 mM ammonium bicarbonate. Peptides were purified using C18 spin columns (150 μm × 100 mm, 3 μm), lyophilized, and reconstituted in 10 μL 0.1% formic acid prior to LC–MS/MS analysis.

#### 2.7.2 UHPLC–MS/MS configuration

Chromatographic separation employed an Easy-nLC 1,200 system coupled to a Q Exactive HF-X mass spectrometer (Thermo Fisher Scientific) through a reversed-phase analytical column (C18, 100 mm × 150 μm, 3 μm; Thermo Fisher Scientific). The mobile phases contained 0.1% formic acid in water (solvent A) or 80% acetonitrile (solvent B). The gradient profile was progressed as follows: 2% B (0–5 min), 8–40% B (5–81 min), 40–95% B (81–83 min), 95% B (83–90 min), 95–2% B (90–95 min), and 2% B (95–100 min) at a 0.6 μL/min flow rate. MS parameters were as follows: Full MS scan: 400–1,200 m/z, 60 k resolution, 3.0 e6 automatic gain control (AGC) of the first-order mass spectrometry; 20% high-energy collision dissociation, 30 k resolution, and 1.0 e5 AGC of the secondary mass spectrometry fragmentation mode.

#### 2.7.3 Data analysis

The raw files were processed using MaxQuant v2.1.3 (Max Planck Institute for Biochemistry, Martinsried, Germany) by searching the UniProt human database.^1^ The results were annotated using the Metascape software,^2^ including Gene Ontology (GO) enrichment analysis and Kyoto Encyclopedia of Genes and Genomes (KEGG) pathway analysis. Differentially expressed proteins (DEPs) were identified using Student’s *t*-test. To account for multiple testing, *p*-values were adjusted via the Benjamini–Hochberg procedure to control the false discovery rate (FDR) at 5%. Proteins with FDR < 0.05 and log2FC > 0.58 were deemed statistically significant. These DEPs were subsequently queried against the Cancer Genome Atlas Cervical Squamous Cell Carcinoma and Endocervical Adenocarcinoma (TCGA-CESC) datasets,^3^ with prognostic associations evaluated by Kaplan–Meier analysis and log-rank testing using the survminer package (v0.4.9) in R.

## 3 Results

### 3.1 Distinct metabolic profiling of CC associated with vaginal microbiota

**Figure 1:**
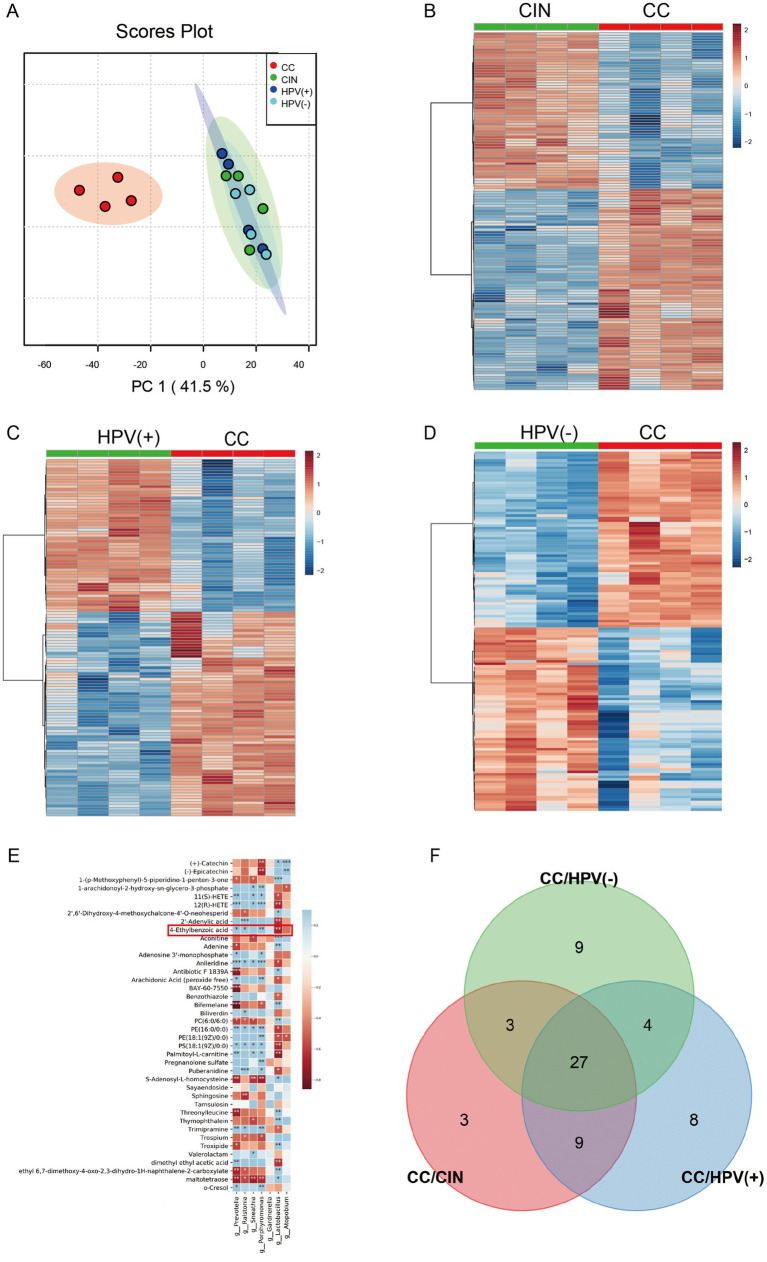
Cervical cancer had a unique vaginal metabolomic profile and was associated with vaginal microbiota. **(A)** Discrimination of groups using principal component analysis. **(B–D)** Hierarchical clustering heat map of differential metabolites in **(B)** CIN patients, **(C)** HPV (+) patients, and **(D)** HPV (−) patients compared with CC patients. And each data point represents pooled samples (*n* = 20/set). **(E)** Spearman correlation analysis between metabolic signatures and vaginal microbiome. **(F)** Venn diagram visualizing the number of shared significantly differential metabolites in CC groups.

Non-targeted metabolomic profiling via UPLC–MS/MS revealed significant intergroup metabolic disparities, as evidenced by principal component analysis (PCA) segregation between the CC group and comparator cohorts—CIN, HPV(+), and HPV(−) groups (Figure 1A). We conducted differential indicator screening and cluster analysis, with VIP > 1 and *p* < 0.05 as the selection screening criteria for selecting differential metabolites. Comparative analyses demonstrated 135, 124, and 143 differentially abundant metabolites distinguishing CC from the CIN, HPV (+), and HPV (−) groups, respectively (Figures 1B–D). Building upon our prior microbiota investigation (19), we performed Spearman’s correlation analysis between these metabolic signatures and vaginal microbial taxa (Figure 1E). CC-elevated metabolites, including 4-EA, PAF C-16, fexofenadine, docosahexaenoic acid ethyl ester, and oxidized glutathione, were positively correlated with CC-enriched genera (*Prevotella*, *Ralstonia*, *Sneathia*, and *Porphyromonas*) and negatively correlated with *Lactobacillus*, which was dominant in HPV (−) controls. This finding demonstrated a close correlation between differential vaginal metabolites and microorganisms.

### 3.2 4-EA is a potential carcinogenic metabolite in CC

Intersectional analysis of shared and unique differential metabolites across the groups was performed using stringent criteria (VIP > 2.0, *p* < 0.05) to screen for more specific differential metabolites. The Venn diagram revealed 27 consensus metabolites that distinguished CC from the other three groups (Figure 1F). The 27 identified metabolites are listed in Table 1. Among these candidate biomarkers, 4-EA was elevated in CC specimens, and correlation analysis (Figure 1E) revealed that 4-EA was negatively correlated with the abundance of beneficial *Lactobacillus* species and positively correlated with the pathogenic bacterium *Prevotella*, suggesting that 4-EA may be a potential carcinogenic metabolite for cervical cancer. ROC analysis demonstrated an AUC of 0.84 (95% CI: 0.23–0.93) for 4-EA, indicating moderate diagnostic utility in cervical cancer detection (Supplementary Figure 1).

**Table 1:** The significant dysregulated metabolites found in the CC group.

Metabolites	Class	VIP^a^			*p*-value^b^	FDR^c^
		CC to CIN	CC to HPV(+)	CC to HPV(−)		
Upregulated						
Eicosapentaenoic acid	Fatty acyls	3.6083	2.3755	2.93	0.014	0.051
LPC 18:2	Glycerophospholipids	3.4926	3.5007	3.5493	≤0.001	0.004
4-Ethylbenzoic acid	Benzene and substituted derivatives	3.265	3.4005	3.5273	0.003	0.018
Nilotinib	Benzene and substituted derivatives	3.175	3.6057	3.396	≤0.001	≤0.001
PC (16:0/0:0)[U]/PC (16:0/0:0)	Glycerophospholipids	3.1344	3.1511	3.2987	≤0.001	0.003
Convallatoxin	Steroids and steroid derivatives	3.1174	2.567	2.1022	≤0.001	≤0.001
Desferrioxamine B	Carboxylic acids and derivatives	3.0654	3.3046	2.7418	≤0.001	0.003
PC (14:0/0:0)	Glycerophospholipids	2.9507	2.6957	2.8383	0.001	0.009
LPC 18:3	Glycerophospholipids	2.8042	2.7306	2.8091	≤0.001	0.004
Arachidonic acid (peroxide free)	Fatty acyls	2.749	2.1903	2.2285	≤0.001	≤0.001
Docosahexaenoic acid ethyl ester	Fatty acyls	2.4747	2.6408	2.2539	≤0.001	≤0.001
LPC 18:1	Glycerophospholipids	2.3284	2.1936	2.3712	≤0.001	0.006
1-arachidonoyl-2-hydroxy-sn-glycero-3-phosphate	Fatty acyls	2.2833	2.8513	2.4087	0.003	0.017
Fexofenadine	Benzene and substituted derivatives	2.264	2.3243	2.3799	≤0.001	0.004
PAF C-16	Glycerophospholipids	2.1696	2.2821	2.329	≤0.001	0.007
Glycocholic Acid	Steroids and steroid derivatives	2.1517	2.4592	2.1564	≤0.001	0.006
Smenospongiarine	Prenol lipids	2.0861	2.7619	2.3637	≤0.001	0.003
Downregulated						
Scalarin	Prenol lipids	3.8072	3.6738	4.7667	0.007	0.031
SB 939	Cinnamic acids and derivatives	3.8015	3.799	3.5592	0.004	0.02
Dihydrokaempferol	Flavonoids	3.5501	3.2527	2.9084	≤0.001	0.004
Notoamide B	Benzopyrans	3.4404	3.5106	3.1179	≤0.001	0.004
2′,6′-Dihydroxy-4-methoxychalcone-4’-O-neohesperid	Flavonoids	3.3072	2.9876	3.1763	≤0.001	0.005
(+)-Catechin	Flavonoids	3.1187	3.0898	3.0687	≤0.001	≤0.001
Catechin 7-arabinofuranoside	Flavonoids	3.1149	2.7434	2.6453	≤0.001	0.007
Bifemelane	Benzene and substituted derivatives	2.3984	2.1317	3.0793	0.01	0.041
(−)-Epicatechin	Flavonoids	2.2541	2.1695	2.045	0.001	0.009
PC (6:0/6:0)	Glycerophospholipids	2.0183	2.3707	2.0247	≤0.001	0.004

a Variable importance in the projection (VIP) was obtained by the orthogonal partial least squares-discriminant analysis(OPLS-DA) model.

b *p*-values were calculated using the student’s *t*-test.

c False discovery rate (FDR) were adjusted via the Benjamini–Hochberg procedure.

### 3.3 4-EA potentiates CC cell proliferation

**Figure 2:**
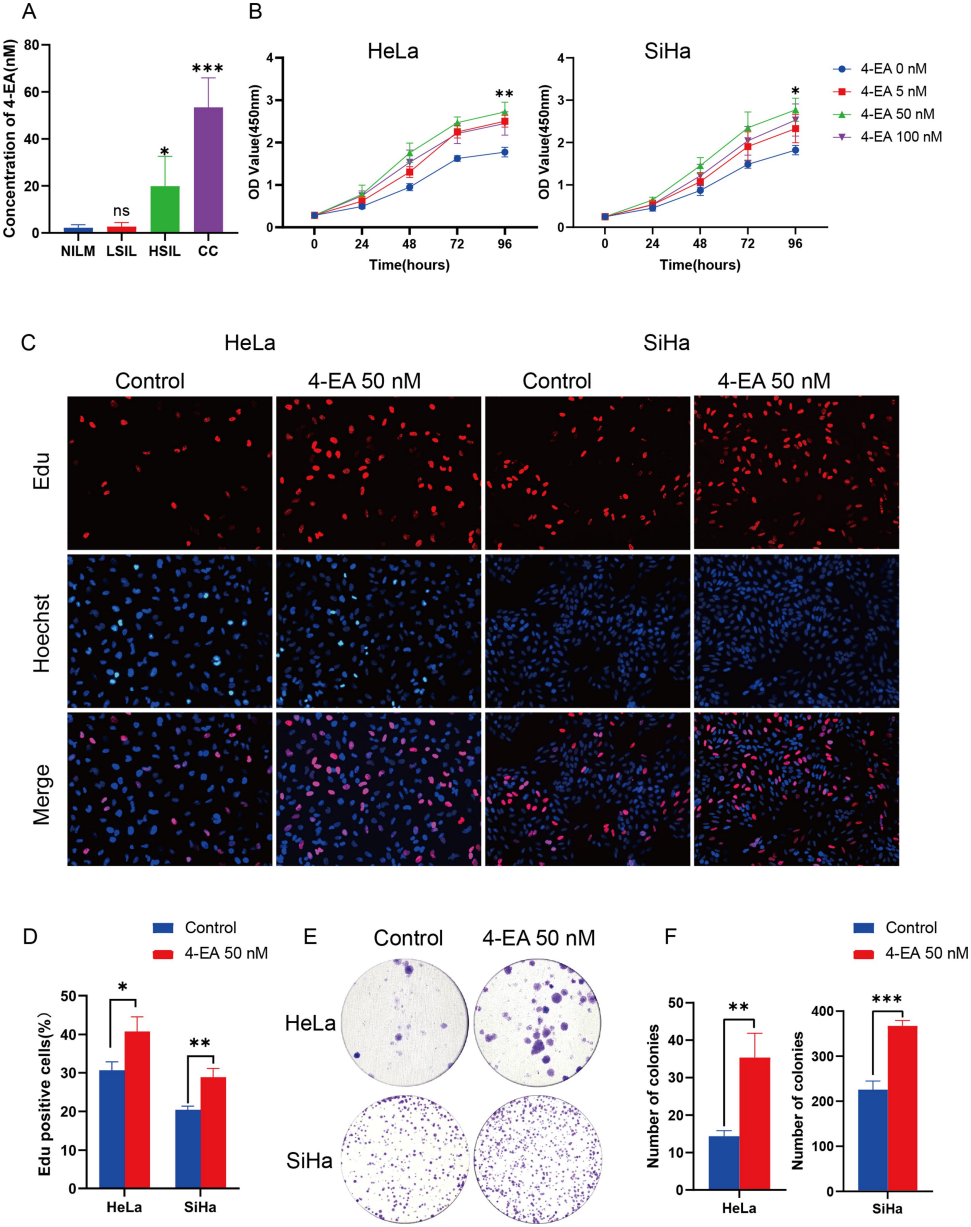
4-EA plays a promoting role in the growth and migration of cervical cancer cells. **(A)** The concentration of 4-EA in vaginal lavage fluids in people with different health conditions by targeted metabolomics. **(B)** Cell viability of HeLa and SiHa cells after 4-EA treatment was detected by CCK-8 assay. **(C,D)** Cell proliferation was examined by EdU assay. **(E,F)** The clone ability of HeLa and SiHa cells was determined by colony formation assay with 4-EA treatment. Data are presented as the mean ± SD. *p < 0.05; **p < 0.01; ***p < 0.001. NILM, negative for intraepithelial lesion or malignancy; LSIL, low-grade squamous intraepithelial lesion; HSIL, high-grade squamous intraepithelial lesion; CC, cervical cancer.

To mechanistically link 4-EA abundance with oncogenic potential, we performed targeted metabolomics profiling of vaginal lavage fluids throughout disease progression stages. Targeted metabolomics revealed the lowest 4-EA concentration in healthy individuals, with a gradual increase observed with the progression of cervical lesions (Figure 2A). Using this clinical concentration range (0–100 nM), we conducted dose–response studies in cervical carcinoma cells (HeLa and SiHa cells). The CCK-8 viability assays demonstrated the proliferative effects of 4-EA, with the most effective stimulation at 50 nM (Figure 2B). This optimal concentration was subsequently employed for functional validation. EdU proliferation and colony formation assays confirmed the pro-proliferative effect of 50 nM 4-EA (Figures 2C–F).

### 3.4 4-EA promotes the migration and invasion of CC cells

**Figure 3:**
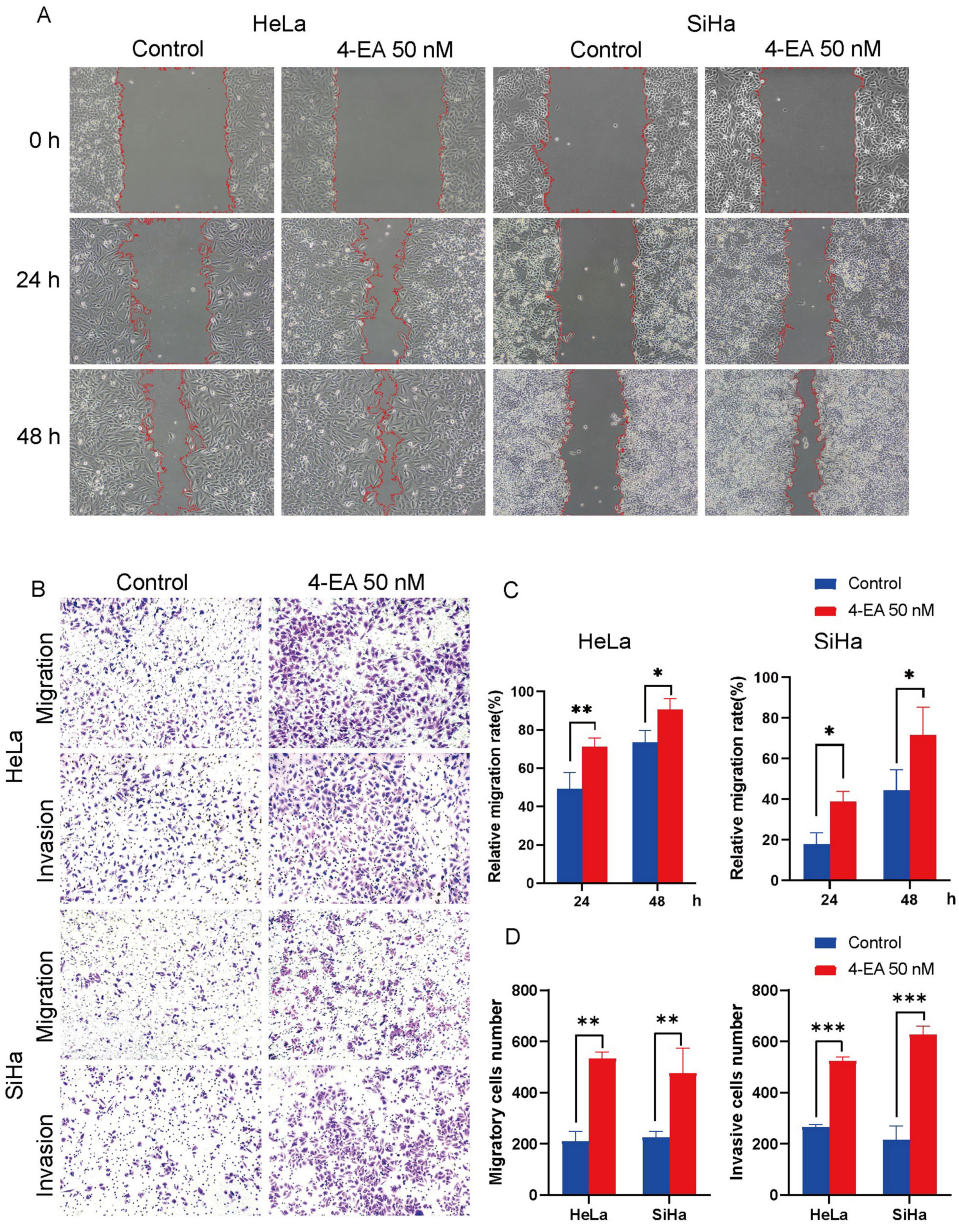
4-EA promoted migration and invasion of cervical cancer cells. **(A,B)** The activity of cell migration was measured by wound healing assay. **(C,D)** The effect of 4-EA on the migration and invasion of cervical cancer cells was detected by transwell migration and invasion assay. Data are presented as the mean ± SD. *p < 0.05; **p < 0.01; ***p < 0.001.

A comparison of the migration rate of 4-EA (50 nM)-treated cell lines and the control group revealed a significantly increased migration rate in the 4-EA group (Figures 3A,B). Furthermore, transwell migration and invasion assays revealed significantly increased numbers of migratory and invasive cells following treatment with 4-EA (Figures 3C,D).

### 3.5 Changes in protein expression in HeLa cells following 4-EA treatment

**Figure 4:**
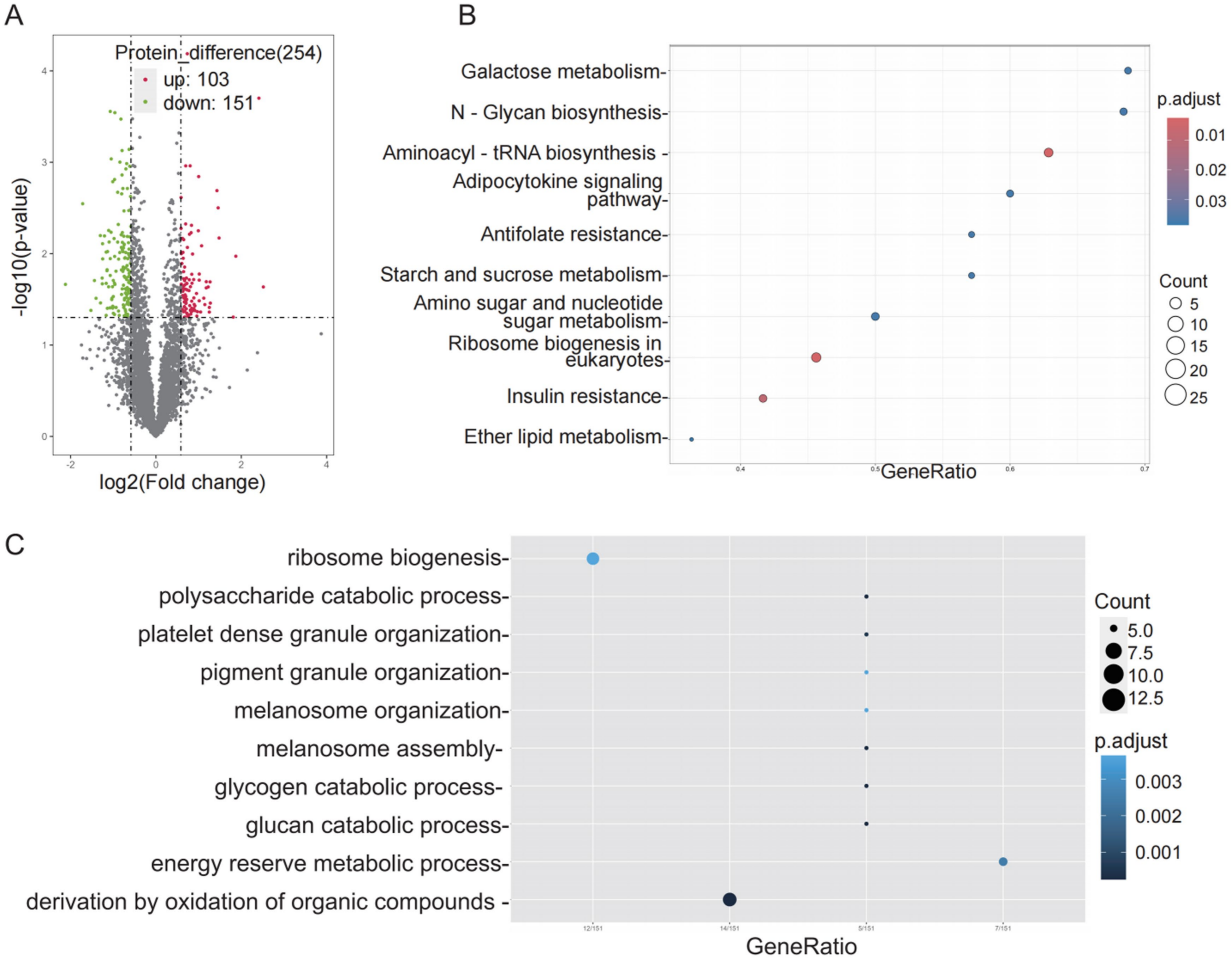
Proteomic analysis of HeLa cells. **(A)** Volcano plot of HeLa cell proteins. The thresholds for *p*-value and |log2FC| were set at 0.05 and 0.58, respectively. FC, fold change of treated group to control group; up, up-regulation; down, down-regulation. Top 10 pathways of differential metabolites for **(B)** KEGG enrichment analysis and **(C)** GO enrichment analysis. Each group included 4 biological replicates.

UHPLC–MS/MS-based proteomic profiling of 4-EA-treated HeLa cells (50 nM, 48 h) identified 254 DEPs compared with untreated controls. Quantitative analysis revealed 103 upregulated proteins (FC ≥ 1.5 and *p* < 0.05, *n* = 4) and 151 downregulated proteins (FC ≤ 1/1.5 and *p* < 0.05, *n* = 4) proteins (Figure 4A). KEGG pathway analysis highlighted 10 perturbed metabolic networks, including galactose metabolism, N-glycan biosynthesis, starch and sucrose metabolism, amino sugar and nucleotide sugar metabolism, and ether lipid metabolism (Figure 4B). Furthermore, GO enrichment analysis demonstrated a significant association of DEPs with the glycogen catabolic process, glucan catabolic process, organic compound oxidation, and polysaccharide catabolic process (Figure 4C).

### 3.6 Key proteins associated with the prognosis of CC

**Figure 5:**
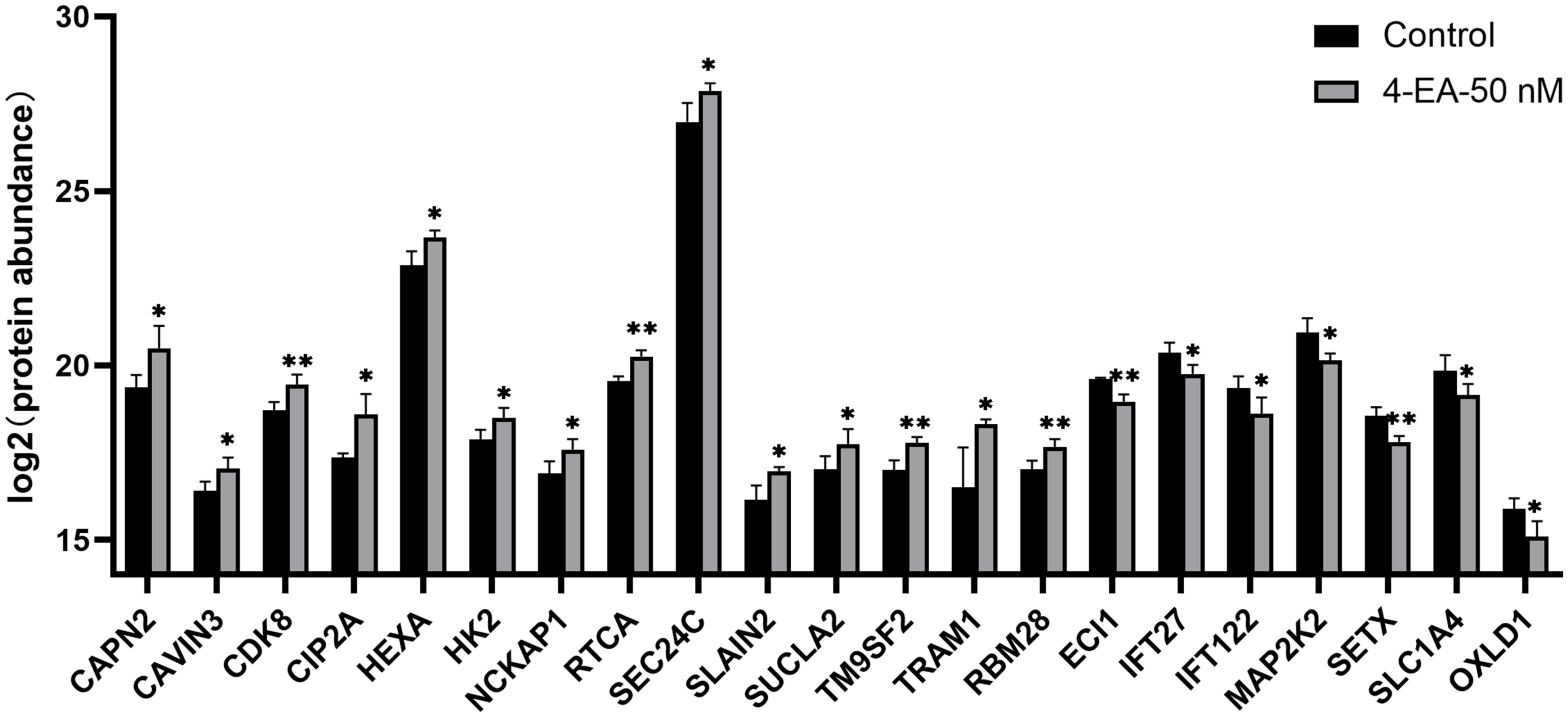
Expression levels of key proteins related to the prognosis of cervical cancer after 4-EA intervention. *p < 0.05; **p < 0.01; ***p < 0.001.

Survival analysis was conducted using TCGA data to evaluate the 254 HeLa-derived DEPs. Notably, 14 upregulated proteins (CAPN2, CAVIN3, CDK8, CIP2A, HEXA, HK2, NCKAP1, RTCA, SEC24C, SLAIN2, SUCLA2, TM9SF2, TRAM1, and RBM28) exhibited adverse prognostic impacts (*p* < 0.05), whereas 7 downregulated proteins (ECI1, IFT27, IFT122, MAP2K2, SETX, SLC1A4, OXLD1) correlated with improved survival (*p* < 0.05). The Kaplan–Meier curves for all 21 candidates are presented in Supplementary Figure 2. The levels of these 21 proteins are shown in Figure 5.

## 4 Discussion

Metabolic reprogramming has emerged as a hallmark of carcinogenesis, and metabolomics provides critical insights into pathogenic mechanisms through precise biomarker identification (20, 21). This approach holds great significance in elucidating cervical carcinogenesis and developing diagnostic and therapeutic strategies. Current descriptive investigations of cervical tissues, serum, urine, and cervicovaginal secretions have confirmed metabolic perturbations during cervical carcinogenesis (6, 22–24). Emerging studies have indicated that vaginal dysbiosis can alter cervicovaginal metabolic landscapes (primarily amino acids, dipeptides, polyamines, and ketone body pathways), potentially affecting the occurrence of CC (25, 26). Furthermore, biogenic amines, glutathione derivatives, and lipid mediators are associated with HPV infection (17). However, although these descriptive studies catalog metabolic shifts, the functional impact and mechanistic interplay of vaginal microbiome metabolites on the host responses remain poorly characterized.

We performed untargeted metabolomic profiling to map the metabolic characteristics of CC cohort to elucidate the functional significance of cervicovaginal metabolites in carcinogenesis. PCA revealed a significant separation trend in the CC metabolic profile compared with the CIN, HPV (+), and healthy cohorts. We conducted the Venn diagram to revealed consensus differentially metabolites that distinguished CC from the other groups. Among these metabolites, elevated levels of PC, LPC, and nilotinib align with established reports of their upregulation in CC (15, 27). However, several metabolites demonstrate previously undocumented associations with cervical carcinogenesis, warranting mechanistic investigation into their pathogenic roles. Our concurrent vaginal microbiota sequencing data demonstrated *Prevotella*, *Ralstonia*, and *Gardnerella* enrichment coupled with Lactobacillus depletion in the CC group (19). These findings suggest associations between microbial community restructuring (*Prevotella* enrichment, *Lactobacillus* depletion) and metabolic dysregulation in cervical carcinogenesis. The marked elevation of 4-EA in CC specimens strongly correlated with *Prevotella* abundance and was inversely associated with Lactobacillus colonization, indicating that 4-EA, as a candidate metabolite, is closely related to vaginal microorganisms. In addition, 4-EA exhibited comparable differential diagnostic performance to established serum biomarkers such as HE4 and SCC-Ag (28). Critically, its non-invasively collected vaginal lavage samples provide logistical advantages over blood-based markers. The diagnostic efficacy was potentially enhanced when combined with HPV testing, suggesting 4-EA has certain synergistic value in clinical screening workflows.

As a benzoic acid derivative, 4-EA belongs to the aromatic carboxylic acid family and occurs naturally occurring in plants and microbial metabolites (29). Benzoic acid alters gut microbial diversity and changes gut barrier function through specific immune responses, nonspecific barrier mechanisms, and microbiota (30). Notably, 4-EA-induced metabolites are involved in steroid hormone biosynthesis (31). Specifically, plasma metabolomics studies have documented age-dependent 4-EA accumulation, with higher levels in older individuals than in younger individuals (32). Benzoic acid and its derivatives are associated with breast and colorectal malignancies (33, 34), while their involvement in CC was previously unreported. Our multi-omics approach firstly reveals 4-EA as the metabolite linked to cervical malignancy, but its mechanistic roles remain to be explored.

To determine the pathophysiological concentrations of 4-EA, we conducted targeted metabolomic quantification of vaginal lavage fluid across clinical cohorts. The CC group exhibited the highest 4-EA levels. Reportedly, 4-EA is associated with a tobacco smoke-induced increase in the permeability of human lung fibroblast membranes, with the effect becoming more pronounced with prolonged exposure and alkyl substitution of the aromatic ring (35). We hypothesized that pathological accumulation of 4-EA was associated with cervicovaginal dysbiosis and 4-EA potentially acted as a cofactor in cervical carcinogenesis. We then conducted *in vitro* experiments using clinically relevant concentrations to explore its biological effects on CC cells. CCK-8 assays revealed the proliferative stimulation of CC cells (HeLa/SiHa) following 4-EA treatment, with the most effective response at 50 nM. Subsequent experiments further validated that 4-EA promotes the proliferation, migration, and invasion of CC cells in vitro. Our research first revealed the promoting effect of 4-EA on CC cells. Notably, although our in vitro studies confirmed 4-EA’s oncogenic effects, direct evidence linking *Prevotella* to 4-EA biosynthesis *in vivo* remains limited. Future studies should explore 4-EA production in gnotobiotic models colonized with clinical *Prevotella* isolates and assess causality via microbiota-depletion experiments.

Carcinogenesis necessitates profound metabolic reprogramming to sustain rapid proliferation and bioenergetic demands, driving critical alterations in metabolic pathways (36). Proteomics analysis is used to directly investigate the possible mechanisms behind the observed phenotypic changes. It showed that the differentially expressed genes were mainly enriched in glycolytic and lipid metabolic pathways, providing new mechanistic insights. Glycolysis is the most widely used to drive various metabolic activities and energy production in tumor cells (37), also lipid metabolism is a key part of tumor energy metabolism (38). This metabolic rewiring aligns with the Warburg effect—a hallmark of malignant progression—while lipid metabolic activation supports membrane biosynthesis for metastatic dissemination (39). It was indicated that 4-EA may play a role in the progression of CC by regulating glucose and lipid metabolism. Subsequently, we combined the TGGA database to conduct survival analysis on 254 DEPs in HeLa cells, identifying 21 prognosis-linked candidates. Among these, 14 adverse prognostic markers that were significantly upregulated after 4-ethylbenzoic acid intervention were identified, and 7 favorable prognostic indicators that were significantly downregulated were identified. Specifically, 4-EA may potentiate glycolytic dependency by upregulating HK2 via HIF-1α stabilization—a mechanism reported for structurally analogous compounds (40). Notably, the suppression of SLC1A4 (a glutamine transporter) suggests 4-EA may rewire glutamine metabolism to favor glutathione synthesis, countering oxidative stress induced by microbial dysbiosis (41). This aligns with prior observations that benzoic acid derivatives deplete intracellular GSH pools in cervical epithelia (42). These key proteins had the potential to become targets for intervention and therapy.

This study identifies *Prevotella*-associated 4-EA as a novel candidate metabolite in cervical carcinogenesis, several limitations warrant acknowledgment. While Spearman correlations suggest microbe-metabolite associations, our analysis cannot resolve whether these relationships reflect direct causation or shared dependencies on unmeasured confounders. Future studies with larger cohorts and metatranscriptomic data are needed to dissect genus-specific contributions. Our targeted validation cohort remains insufficient for clinical translation. While our pooled sample design enhanced detection sensitivity for low-abundance metabolites, it inherently precluded individual-level correlation analyses between clinical variables and metabolic signatures—a necessary trade-off that future studies with larger cohorts should address. We propose a multicenter validation initiative to be conducted over 3 years across six tertiary hospitals in high-risk Chinese provinces (Guangdong, Henan, Sichuan; total catchment area >50 million). This will enroll 1,200 participants (200/site) for standardized sample collection and centralized UPLC–MS/MS analysis, with stratification by HPV subtype, menopausal status, and socioeconomic factors. This expanded cohort is essential to verify the diagnostic robustness of 4-EA across diverse populations and confounding variables. In addition, proteomics revealed 4-EA regulated glycolytic and lipid metabolic pathways, the precise molecular triggers remain unresolved. The 21 key proteins identified from proteomic analysis require orthogonal verification (Western blot and qPCR) *in vitro* and vivo models, and mechanistic insights into 4-EA’s metabolic reprogramming require deeper interrogation.

## 5 Conclusion

Cervical carcinogenesis displays a distinct cervicovaginal metabolic signature, with microbiome-derived metabolites actively contributing to the malignant progression. Our multi-omics investigation identified 4-EA as a *Prevotella*-associated candidate metabolite, with a significant increase in patients with CC compared with the healthy controls. Furthermore, functional validation revealed that 4-EA promotes the proliferation, migration, and invasion abilities of CC cells and regulates their protein profile, highlighting 4-EA as a non-invasive biomarker. Additionally, proteomic profiling and TCGA survival analysis identified 21 prognosis-linked targets, which may serve as potential targets for the intervention and treatment of CC. In this study, 4-EA was found to be a candidate metabolite related to microorganisms, which has the potential to serve as a biomarker for recognizing CC and provides novel mechanistic insights into CC intervention strategies.

## Data Availability

The metabolomics data have been deposited to MetaboLights repository with the study identifier MTBLS12730. The proteomics data have been deposited to the ProteomeXchange Consortium via the iProX partner repository with the dataset identifier PXD066243.

## Footnotes

^1^ https://www.uniprot.org/

^2^ https://metascape.org

^3^ https://portal.gdc.cancer.gov/
