# Supplementary material for: Multi-omics profiling reveals the role of 4-ethylbenzoic acid in promoting proliferation and invasion of cervical cancer
Source: Front Med (Lausanne). 2025 Oct 13;12:1591531. doi: 10.3389/fmed.2025.1591531 (PMC12557335; doi:10.3389/fmed.2025.1591531)
Supplement: Supplementary file 1 [file Supplementary_file_1.docx]

Supplementary Material

## Supplementary table 1

**Supplementary Table 1** General characteristics of participants in targeted metabolomics.

| Characteristic | HPV(-)(n=8) | HPV(+) (n=8) | CIN(n=8) | CC (n=8) | P^1^ |
| --- | --- | --- | --- | --- | --- |
| HR-HPV(±) | - | + | + | + |  |
| Age (years) | 44.6 (29-56) | 38.6 (26-52) | 32.0 (23-49) | 45.3 (36-62) | 0.056 |
| BMI (kg/m2) | 23.57 (17.67-30.85) | 23.32 (19.98-30.02) | 19.68 (16.44-22.83) | 22.50 (17.63-27.34) | 0.078 |
| Menopause | 3 (37.5) | 4 (50) | 1 (12.5) | 4 (50) | 0.259 |
| Smoking history | 2 (25) | 1 (12.5) | 3 (37.5) | 2 (25) | 0.91 |
| Alcohol drinking history | 4 (50) | 3 (37.5) | 6 (75) | 4 (50) | 0.754 |

**Notes:** Data are presented as averages (range) and numbers (%). ^1^One-way analysis of variance (ANOVA) was used for continuous variables, and the Fisher's exact test was used for categorical variables.

## Supplementary Figure 1

*
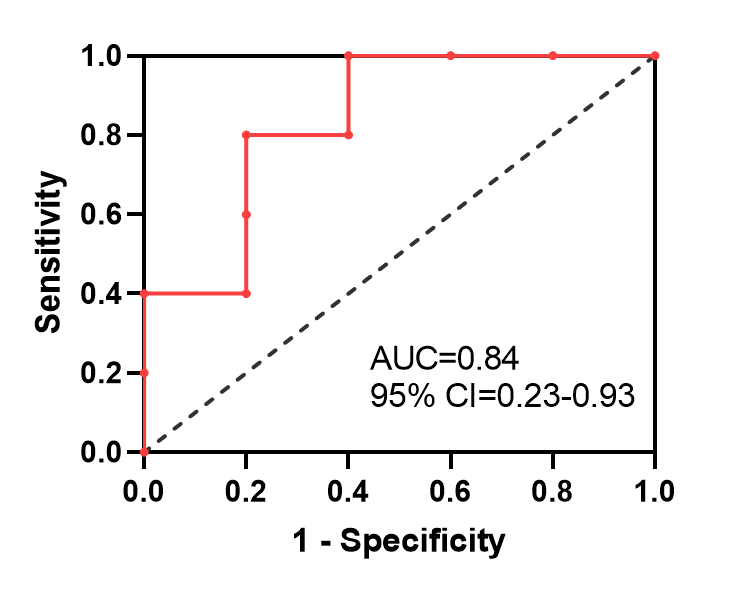
*

*Supplementary Figure 1. RO*C analysis for 4-EA.

## Supplementary Figure 2

##
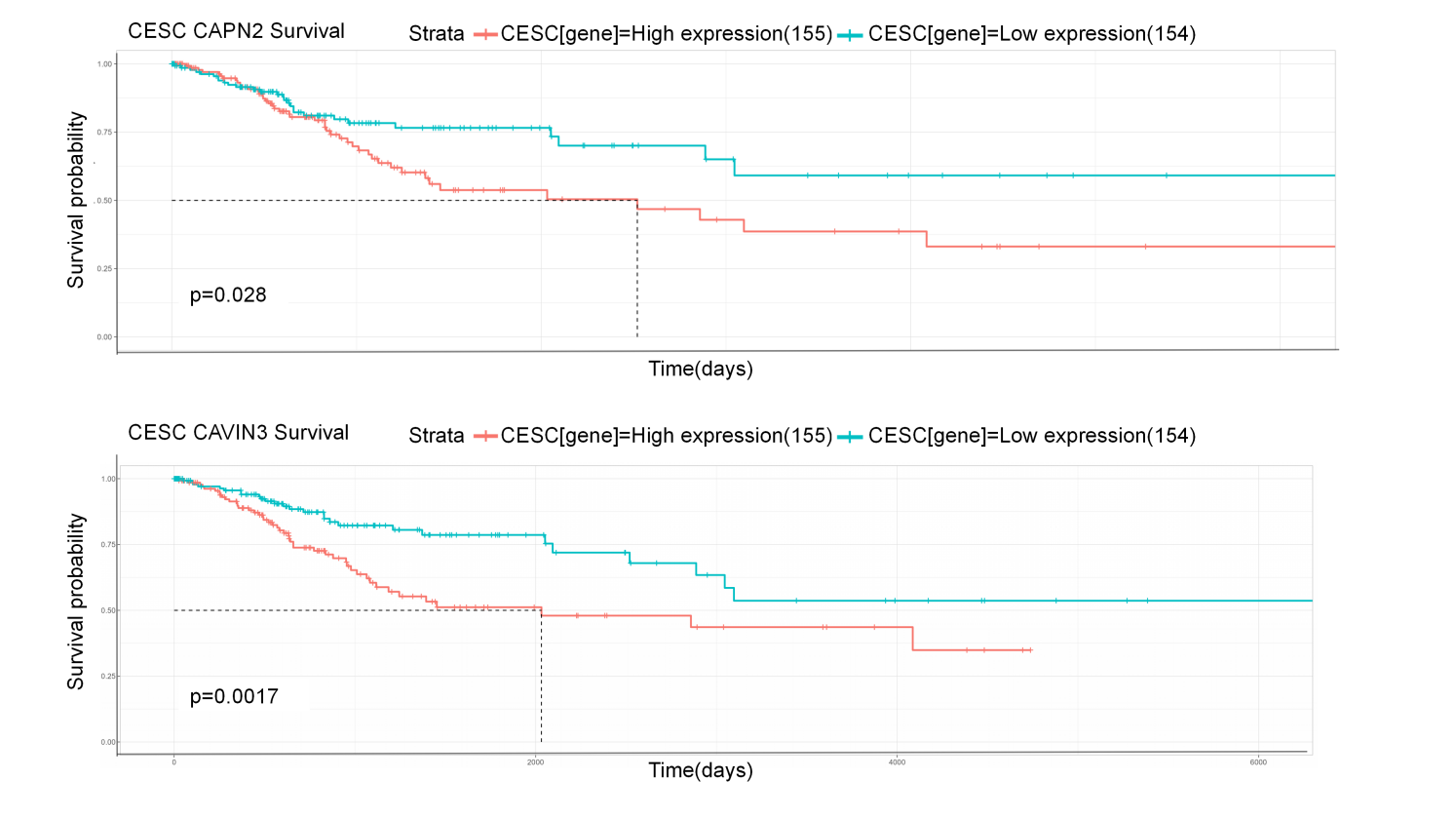

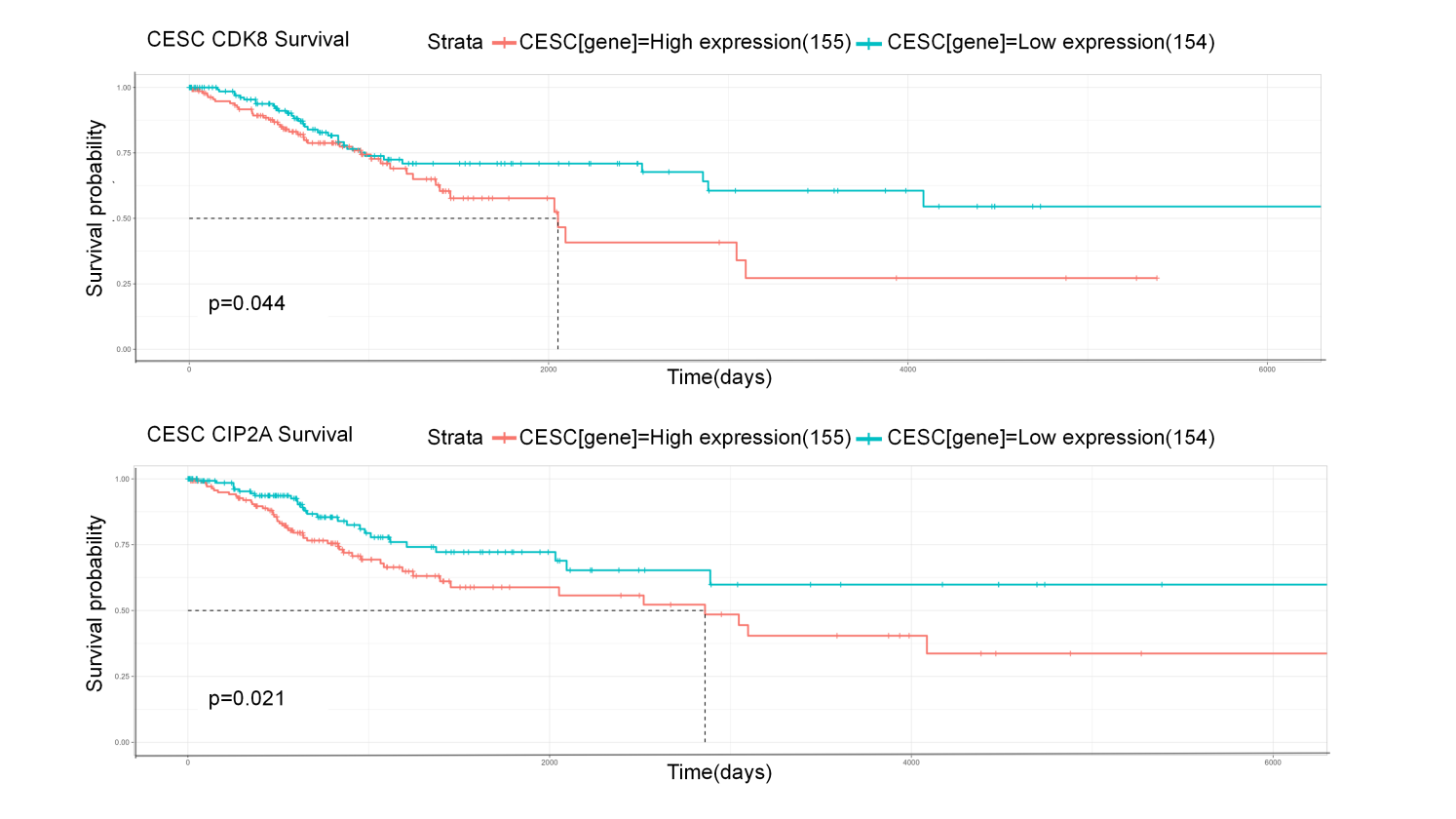


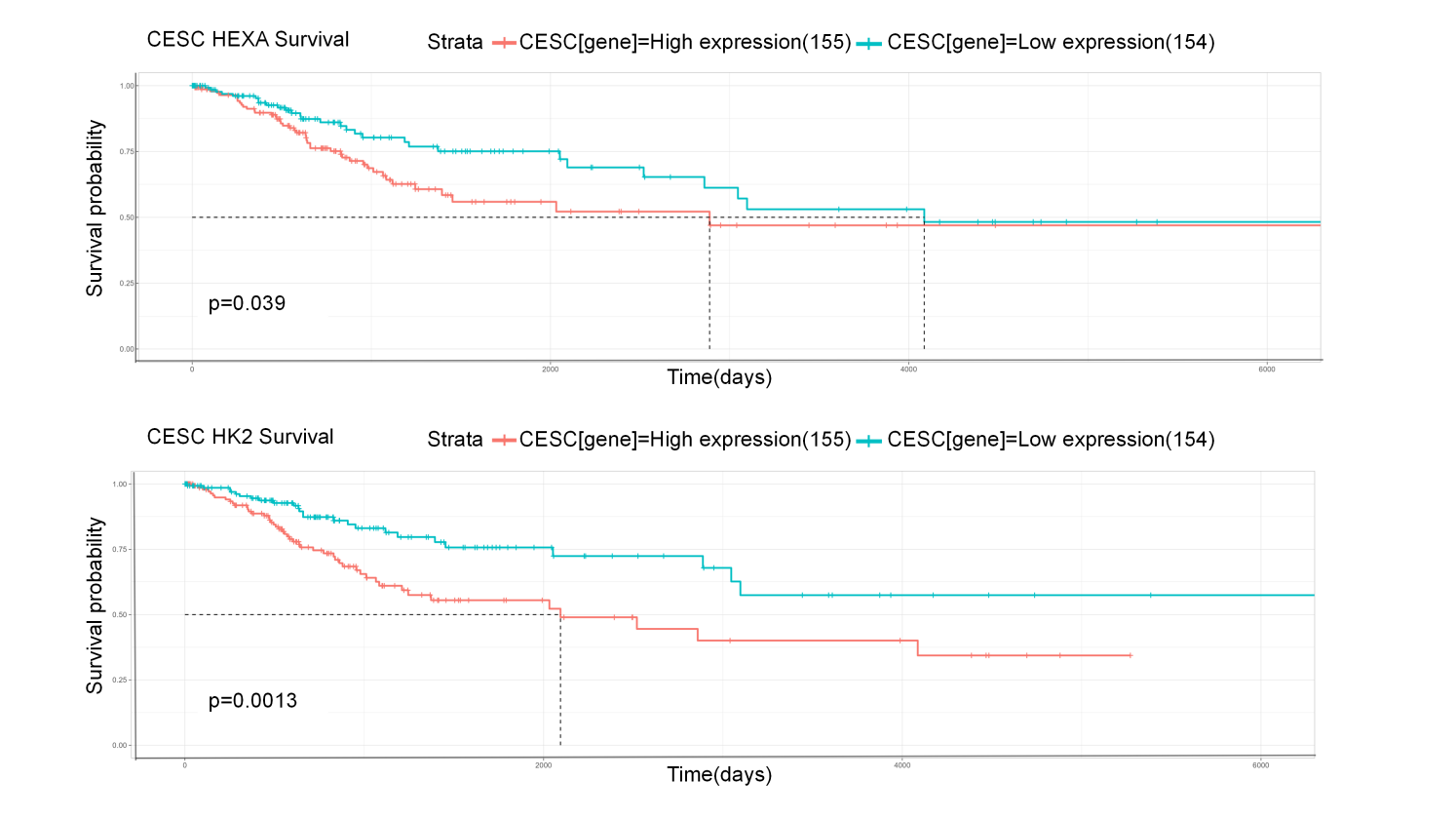


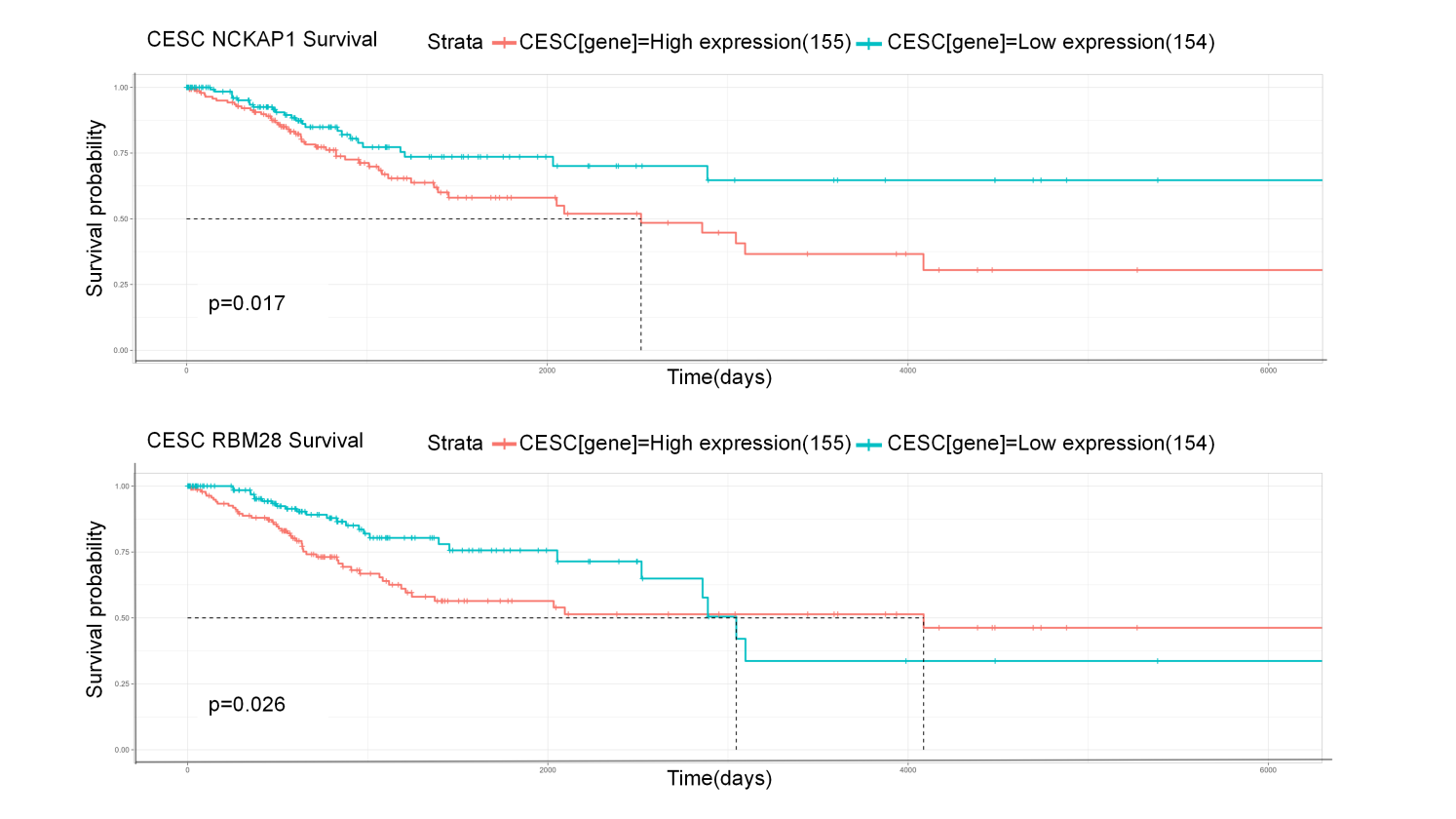


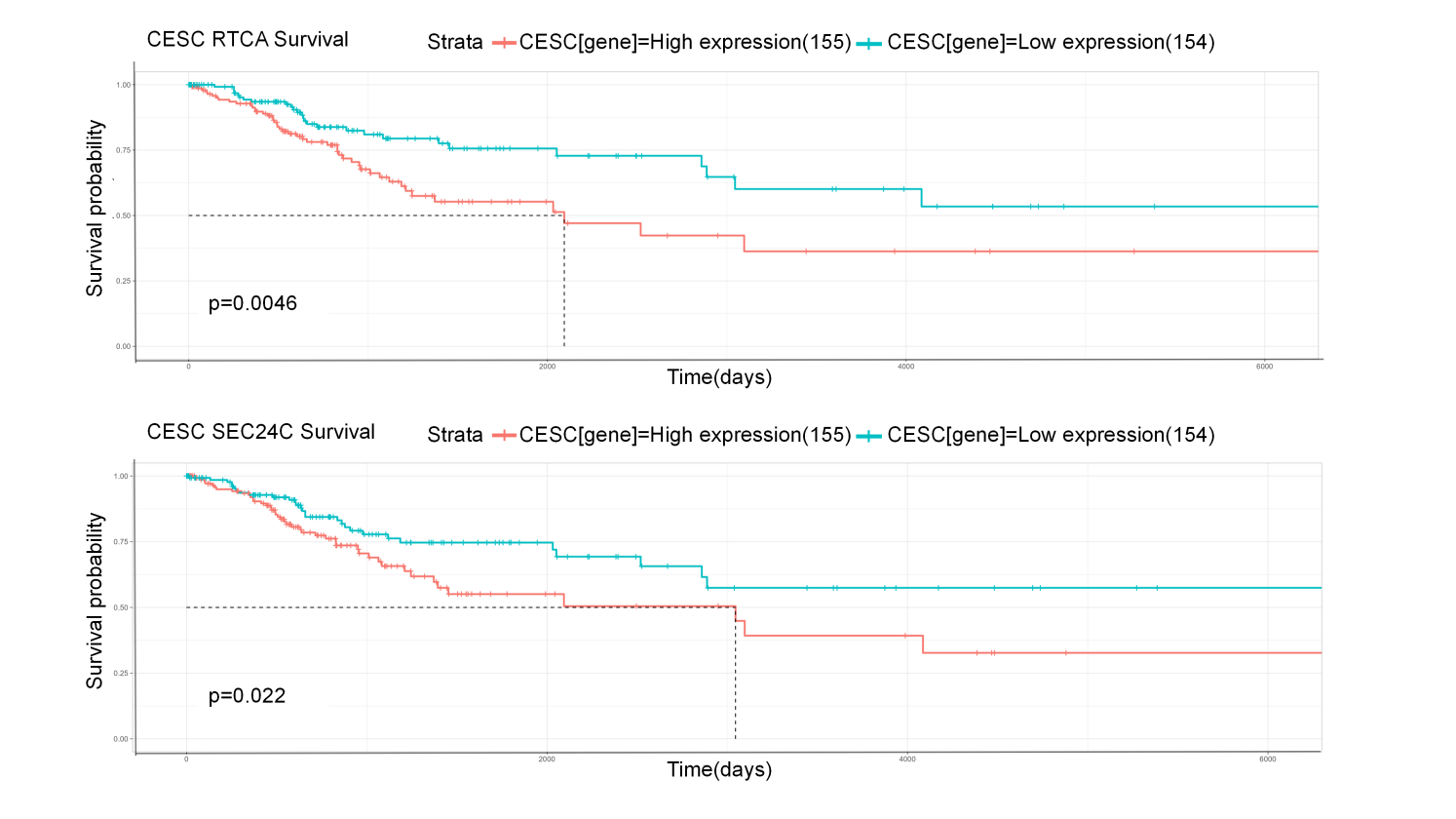


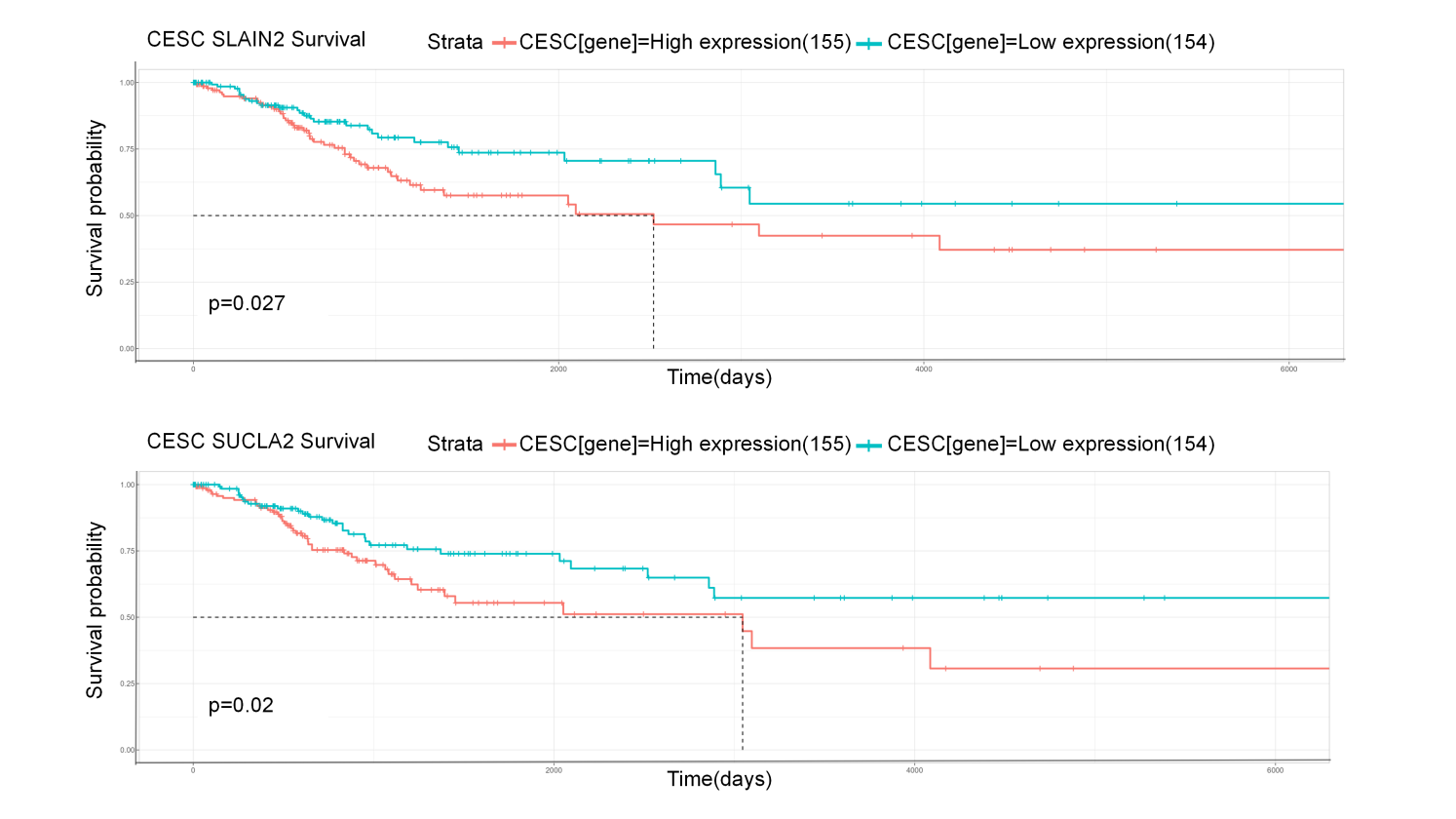


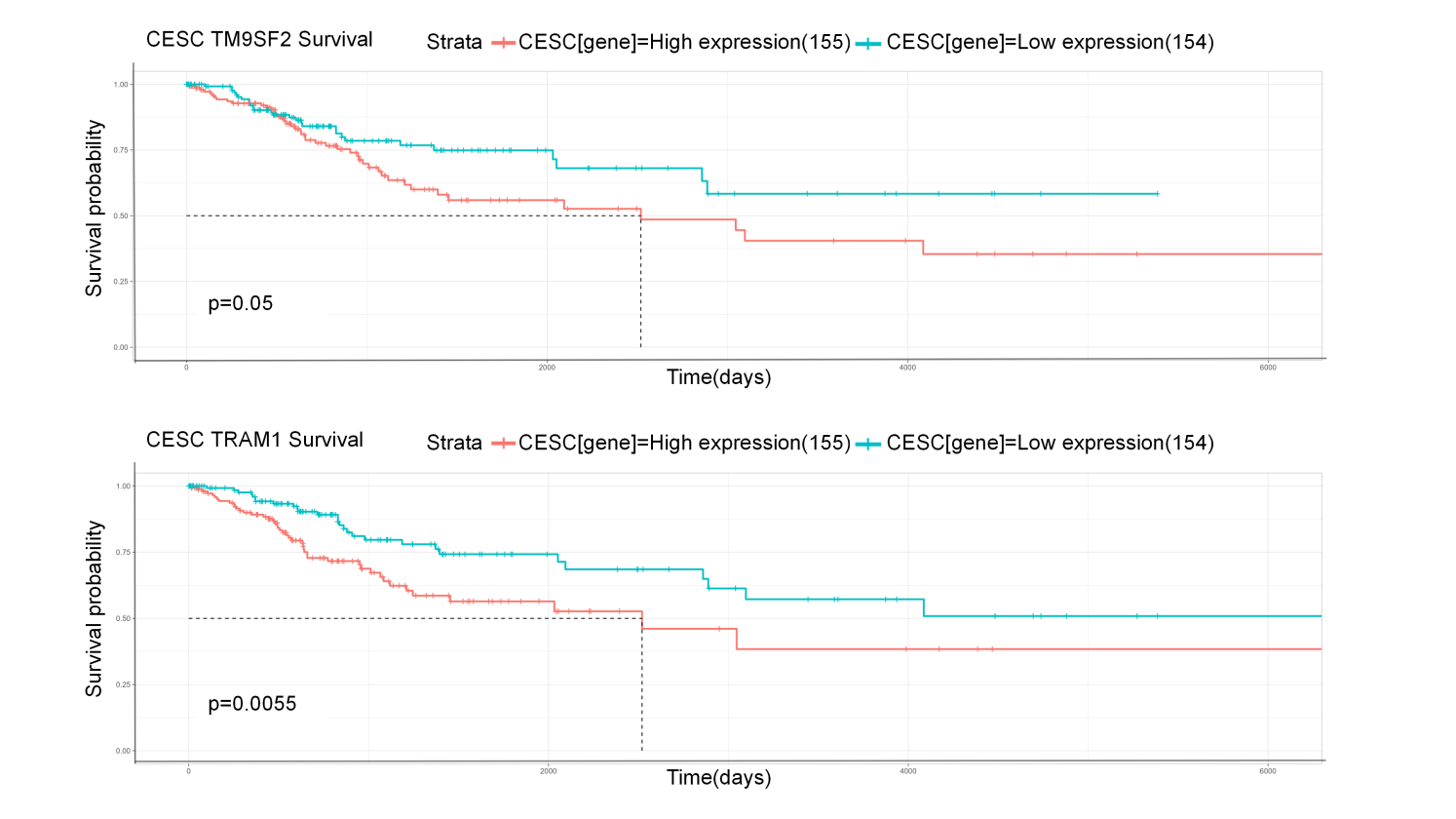


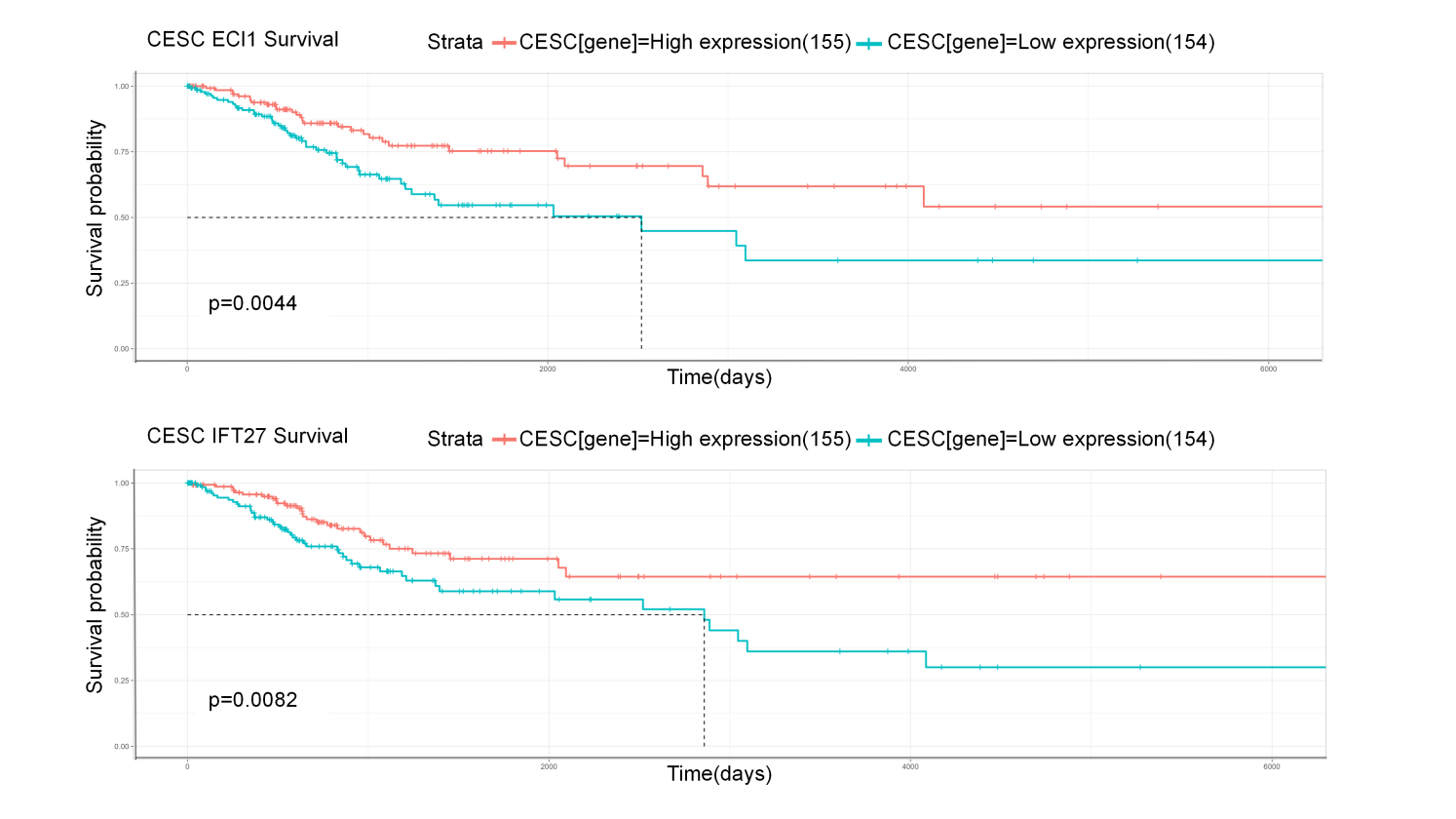


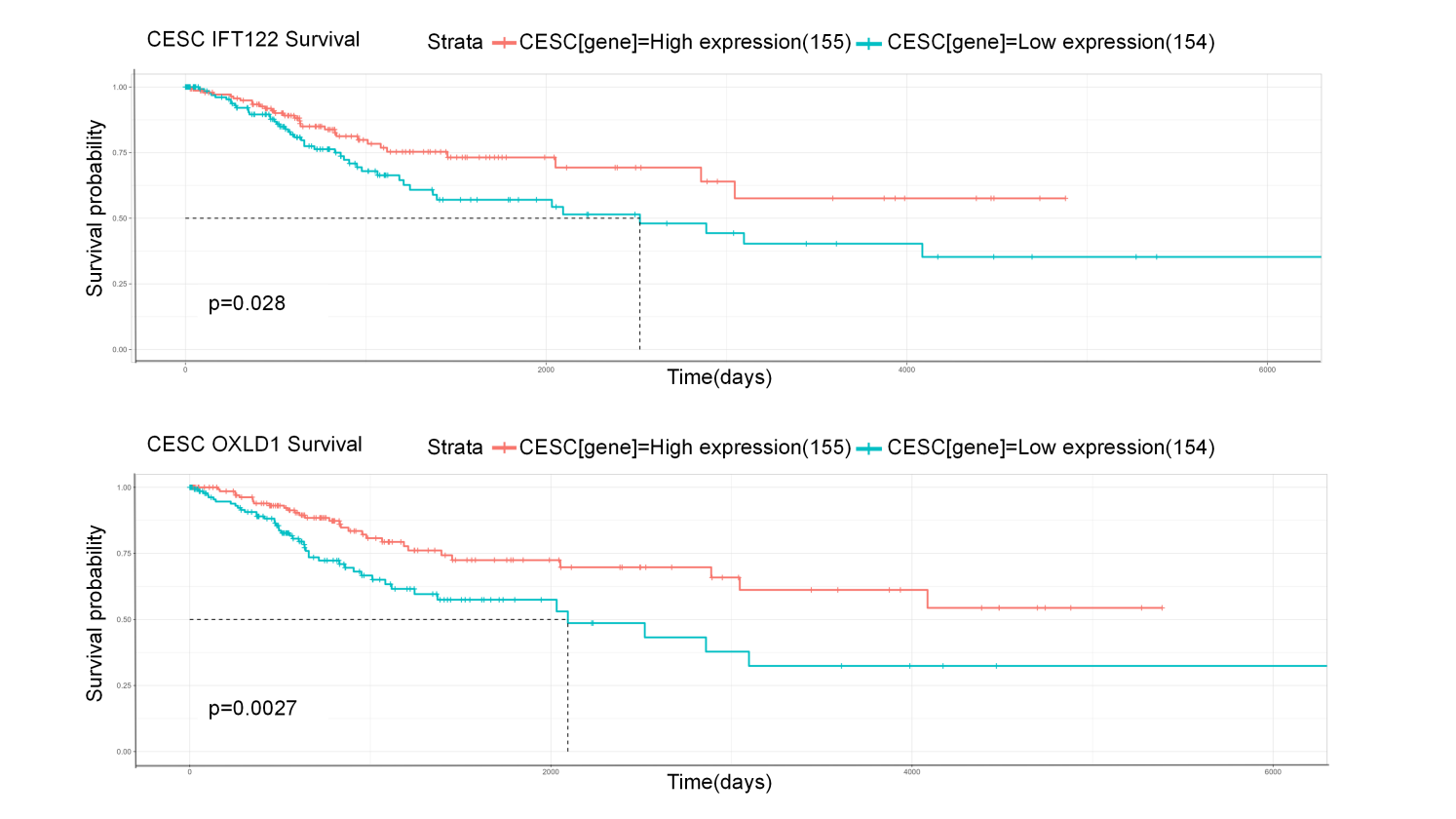


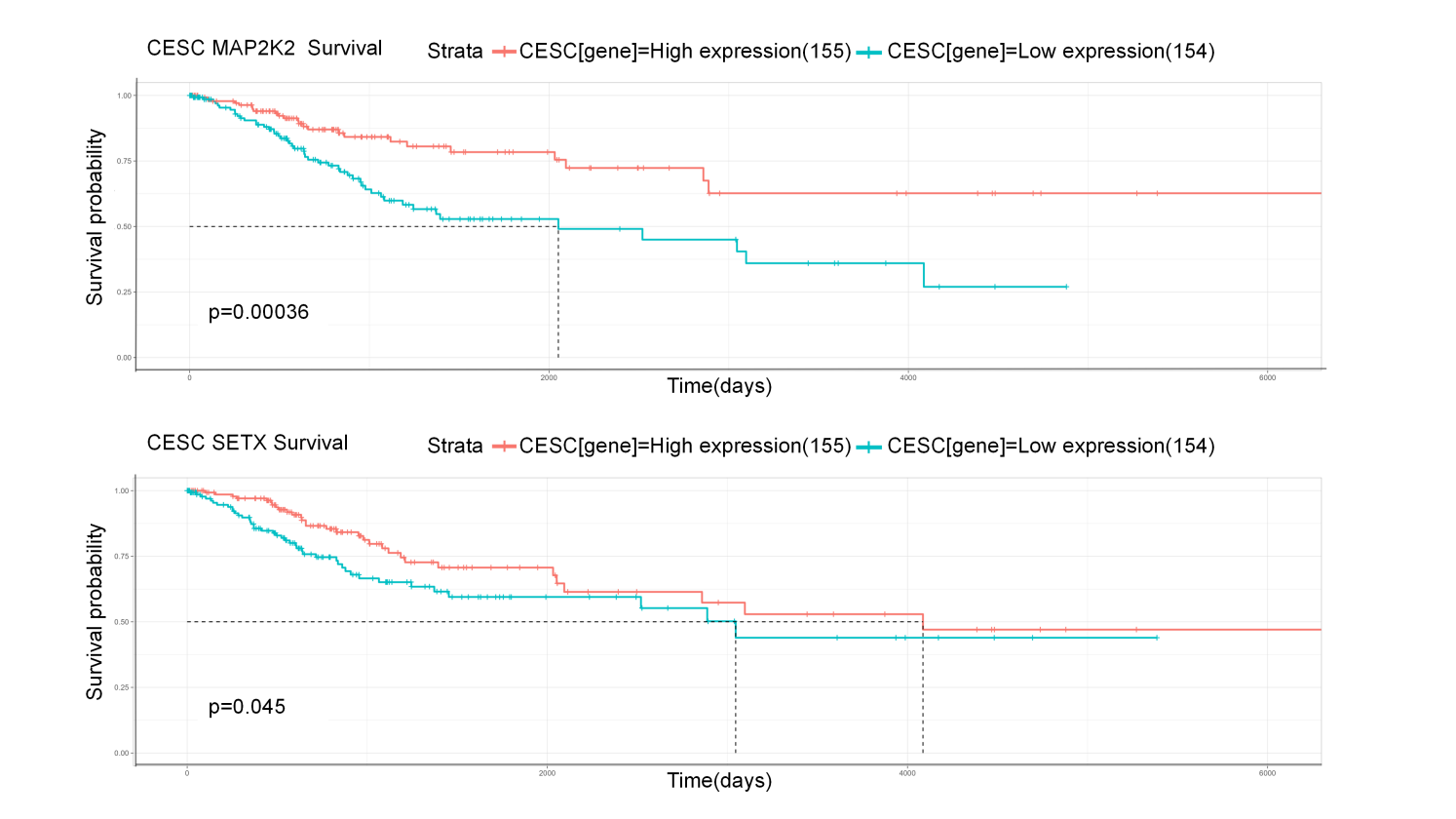


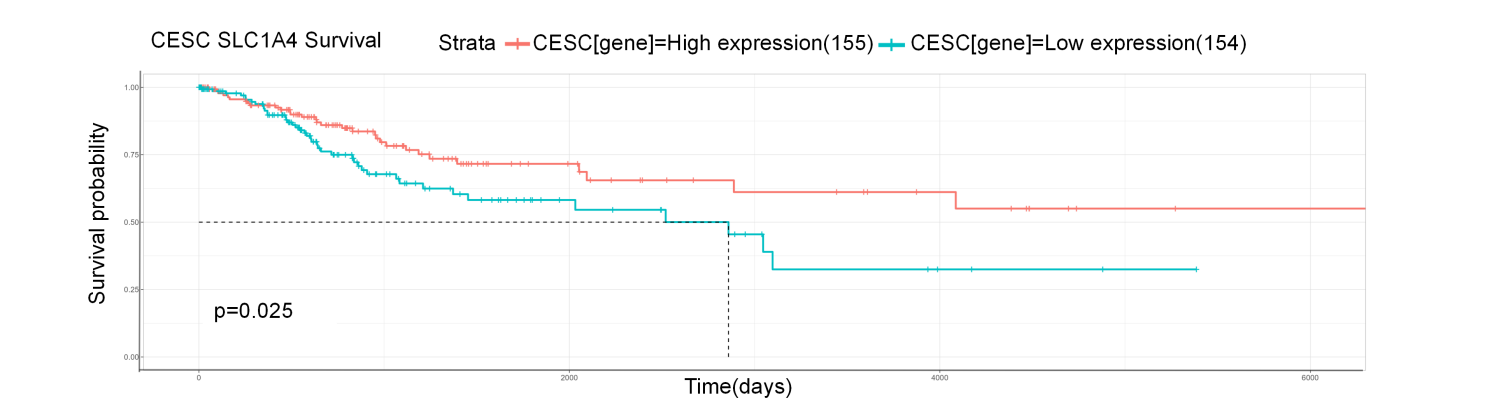


**Supplementary Figure 1.** The Kaplan–Meier curves for all 21 candidates are presented, including 14 upregulated proteins (CAPN2, CAVIN3, CDK8, CIP2A, HEXA, HK2, NCKAP1, RTCA, SEC24C, SLAIN2, SUCLA2, TM9SF2, TRAM1, and RBM28) exhibited adverse prognostic impacts (P < 0.05), and 7 downregulated proteins (ECI1, IFT27, IFT122, MAP2K2, SETX, SLC1A4, OXLD1) correlated with improved survival in atients with CC.
